# Diversity, Co-Occurrence, and Nestedness Patterns of Sand Fly Species (Diptera: Psychodidae) in Two Rural Areas of Western Panamá

**DOI:** 10.3390/insects12020113

**Published:** 2021-01-28

**Authors:** Chystrie A. Rigg, Milixa Perea, Kadir González, Azael Saldaña, José E. Calzada, Yan Gao, Nicole L. Gottdenker, Luis Fernando Chaves

**Affiliations:** 1 Departamento de Investigación en Parasitología, Instituto Conmemorativo Gorgas de Estudios de la Salud (ICGES), Ciudad de Panamá 0816-02593, Panama; mperea@gorgas.gob.pa (M.P.); kgonzalez@gorgas.gob.pa (K.G.); asaldana@gorgas.gob.pa (A.S.); jcalzada@gorgas.gob.pa (J.E.C.); 2 Centro de Investigación y Diagnóstico de Enfermedades Parasitarias (CIDEP), Facultad de Medicina, Universidad de Panamá, Ciudad de Panamá 4 3366, Panama; 3 Centro de Investigaciones en Geografía Ambiental (CIGA), Universidad Nacional Autónoma de México (UNAM), Morelia 58190, Michoacán, Mexico; ygao@ciga.unam.mx; 4 Department of Pathology, School of Veterinary Medicine and Center for the Ecology of Infectious Diseases (CEID) University of Georgia, Athens, GA 30602, USA; gottdenk@uga.edu; 5 Instituto Costarricense de Investigación y Enseñanza en Nutrición y Salud (INCIENSA), Tres Ríos, Cartago 4-2250, Costa Rica

**Keywords:** diversity, sand fly, land use change, seasonal weather, leishmaniasis, Panamá

## Abstract

**Simple Summary:**

Sand flies are insects that can transmit the parasites causing leishmaniasis, a major neglected tropical disease. In the Americas, these insects are highly diverse, and unlike what is observed for other vector-borne diseases, many species co-occur in sites where this disease affects human populations. Here, we present results from a two-year-long study where we study how the number of species changes in two rural areas of Western Panamá with different land use cover and through the dry and wet seasons. We found that species number increased during the wet season and in plots with higher natural forest cover and that species number decreased in both areas in plots when the forest cover decreased, with some species changing through the seasons, and some species disappearing when comparing the sand fly faunas of the most forested with less forested plots. However, our results suggest that seasonality, or the change from a dry to rainy season, can be a more important driver of the number of species locally observed in the studied areas.

**Abstract:**

Cutaneous Leishmaniasis transmission in the New World is observed in areas with rich sand fly species’ faunas. The diversity and composition of sand fly species can change in response to seasonal weather and land use changes. Here, we present results from a two-year-long study where we collected, using Centers for Disease Control (CDC) light traps, sand flies from two rural areas, Las Pavas (LP) and Trinidad de las Minas (T) in western Panamá. Over 710 trap-nights, we collected 16,156 sand flies from 15 genera and 35 species. We identified 34 species in T, and the most abundant species collected was *Nyssomyia trapidoi* (Fairchild and Hertig, 1952) (n = 2278, 37%), followed by *Psychodopygus panamensis* (Shannon, 1926) (n = 1112, 18%), and *Trichopygomyia triramula* (Fairchild and Hertig, 1952) (n = 1063, 17%). In LP, we identified 26 species, and the most abundant species collected were *Ty. triramula* (n = 4729, 48%), and *Ps. panamensis* (n = 3444, 35%). We estimated a higher species’ richness in T (Chao2 ± S.E.: 36.58 ± 3.84) than in LP (27.49 ± 2.28). In T, species’ richness was significantly higher in the rainy season, but no seasonal differences were observed in LP. Species’ assemblages were nested in the two areas. Phlebotomine sand fly species’ abundance increased at the two sites during the rainy season. Our data suggest that seasonality is more important than land use as a factor driving sand fly species’ diversity at the studied sites.

## 1. Introduction

Leishmaniasis is a vector-borne disease caused by flagellated protozoan parasites of the genus *Leishmania* Ross, 1903 and transmitted by the bite of infected female phlebotomine sand flies. There are three main clinical forms of the disease, cutaneous (CL), mucosal (ML), and visceral leishmaniasis (VL) [1]. An average of 55,000 cases of CL and ML and 3500 cases of VL are reported every year in the Americas, with an average case fatality rate of 7%. In this region, CL is considered endemic in 18 out of 20 countries, and 27% of cases occur in international border areas [2]. In Panamá, approximately 37,576 cases of CL have been reported from 2000 to 2019, with the peak of cases registered in 2006 (3774 cases) and 2010 (3221 cases). Provinces of Bocas del Toro (29%), Cocle (16%), Panamá Oeste (16%), and Colón (11%) are the areas with the highest transmission and infection risk [3].

The principal etiologic agent of cutaneous leishmaniasis in Panamá is *Leishmania (Viannia) panamensis* Lainson and Shaw, 1972, and its main reservoir is the Hoffmann’s two-toed sloth, *Choloepus hoffmanni* Peters, 1858 (Pilosa: Megalonychidae) [4,5]. Parasite transmission is attributed to the bite of infected female sand flies (Psychodidae: Phlebotominae) on wild and domestic animals [1,2,6].

Forest fragmentation and deforestation could alter the transmission of vector-borne diseases, such as leishmaniasis since this forest cover and structure alter the distribution, diversity, and co-occurrence of vector, reservoirs, and human hosts [7,8]. Moreover, changes in sand fly species’ diversity and abundance can occur in response to seasonal weather variations, such as rainfall, temperature, and relative humidity fluctuations [9,10,11].

The diversity of sand fly species in Panamá is high, with 76 described species [12]. These sand flies inhabit a range of different environments, and *Leishmania* transmission depends on the overlap between vector, mammalian reservoir hosts, and human populations [5,13,14]. In some regions, the presence of vector species in the human environment may be associated with the emergence of autochthonous cases of leishmaniasis [15,16], as occurs in the Western Panamá Province, where CL is widespread [3]. It is of epidemiological interest to understand how both land use and seasonality impact sand fly species’ diversity and composition. It is also important to understand if species are nested across land use types, with a core set of epidemiologically important species persisting through seasons and land use types that can sustain year-round CL transmission [17,18,19].

A unique characteristic of phlebotomine sand flies in the Neotropics is the co-occurrence of several medically important sand fly species with proven vectorial capacity and competence at endemic leishmaniasis transmission foci [20,21,22,23,24,25]. Nevertheless, relatively little research has been done to study the structure of these sand fly communities regarding their diversity patterns, i.e., the change in species’ composition across an environmental gradient [26]. In particular, understanding diversity patterns can be useful to predict species that are likely to become vectors, given that some species might have similar ecological patterns to those currently recognized as dominant vectors [27]. Furthermore, the co-occurrence of vector species with species without medical importance can be an indicator of the likelihood of disease transmission, as it has been reported that an increased vector diversity is associated with lower infection rates in dominant vector species [28,29].

Null model tests of species’ co-occurrence and nestedness are ecological tools that have become increasingly useful to study diversity patterns. The conceptual basis of these methods is to estimate metrics measuring co-occurrence and/or nestedness using field data and compare this result with distributions of the same metric generated by simulations fulfilling certain constraints/assumptions [30,31,32].

Here, we present the results from a two-year study where we sampled sand flies in two rural areas in western Panamá across a land use gradient. Co-occurrence and nestedness null models and multisite metrics analysis were used to evaluate if patterns of species’ composition were affected by the distance between sampling plots [26]. We also evaluated the impact of seasonality and land use degradation on sand fly species’ diversity.

## 2. Materials and Methods

### 2.1. Study Site

**Figure 1:**
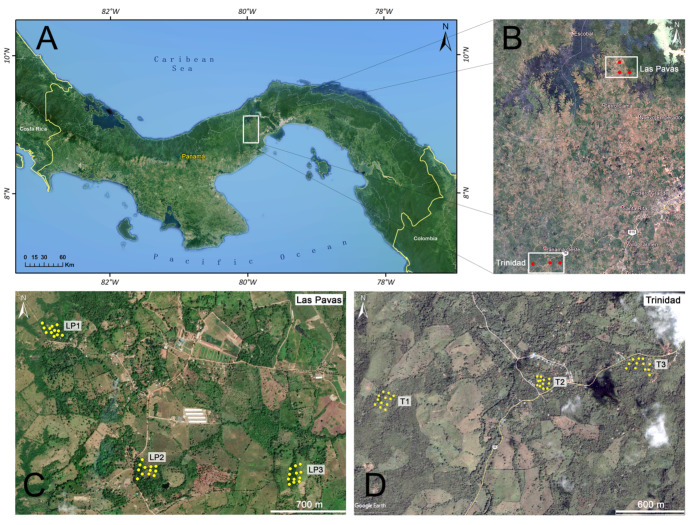
Geographic location of the communities Trinidad de las Minas (T) and Las Pavas (LP) in Panamá Oeste Province. (**A**) Map of Panamá and general study location. (**B**) Geographic location of study sites relative to each other. (**C**) Las Pavas (LP) sampling locations: peridomicile plot (LP1), partly wooded plot (LP2), wooded plot (LP3). (**D**) Trinidad de las Minas (T) sampling locations: wooded plot (T1), peridomicile plot (T2), partly wooded plot (T3). Numbers in the plot labels were assigned from west to east. Wooded plots are covered by primary forest, while peridomicile and partly wooded plots are a mixture of secondary vegetation and standing trees, the peridomicile plots being within a 100m radius from households. The images in this figure are courtesy of Google and were accessed from Google Earth, version 7.3.3.7786 (earth.google.com/web/). The figure was elaborated with Arcmap (ESRI 2011) and CorelDRAW, version X7 (17).

The study took place in an area west of the Panamá Canal (Figure 1A), consisting of protected late secondary moist tropical forest adjacent to the canal, flanked by a mosaic of disturbed habitat types whose natural vegetation is lowland moist tropical forest [33]. These highly disturbed areas consist of patches of forest remnants primarily located in riparian areas, habitat patches in early phases of forest regeneration (abandoned pasture), cattle pasture, and human areas dominated by housing units [34]. The Panamá Canal Watershed (PCW) is located in the central part of the country and has a total area of 339,000 hectares; some 157,000 hectares (or 47% of the total) are covered by forests. Almost 70% of this forested area is within the Chagres, Altos de Campana, Soberanía, Camino de Cruces, and the Barro Colorado Natural Monument. Outside of the protected areas, however, forest remains are made up of small scattered patches and gallery forests that are gradually being lost [35].

The PCW area is distributed among 3 provinces, Western Panamá, Panamá, and Colón, and comprises 7 districts: Panamá, Arraiján, La Chorrera, Capira, Colón, Portobelo, and a very small portion of Chagres. This study took place in two settlements in central Panamá (Figure 1A): Las Pavas (LP) and Trinidad de las Minas (T), located in Western Panamá Province (Figure 1B), west of the Panamá Canal (Figure 1A). The climate of the region varies with its topography and global climatic phenomena, such as “El Niño Southern Oscillation”, that are independent of land use [36].

Las Pavas (9°6′15″ N, 79°53′9″ W), is located in the Panamá Canal watershed, in fragmented forests in the tropical rainforest life zone [33], within Amador corregimiento (municipality), in La Chorrera district. The mean annual temperature in this region changes depending on land use, with an annual average of 26 °C inside the primary forest, and 32 °C to 42 °C in open fields [37]. Annual precipitation varies between 2000 and 2500 mm, with moderate winds of about 10 km/h, and soils of low fertility [38], with two well marked seasons, one dry from January to May and one rainy from June to December [39]. This community is scattered across a landscape whose elevation varies between 50 and 156 m. The primary forest has been extensively transformed into pastures, but a few isolated forest patches remain through the landscape.

Trinidad de las Minas (8°46′32″ N, 79°59′45″ W), located within Cacao corregimiento (municipality), in Capira district, is an area of endemic cutaneous leishmaniasis transmission [13]. The town core is located at an elevation of 250 m, with a mean annual temperature of 26 °C and annual rainfall varying between 28 and 570 mm. The dry season is from January to March, the rainy season from April to December [40]. T is located in a humid tropical forest zone with a sub-equatorial climate. The annual temperature averages from 26.5 to 27.5 °C in the lowlands (<20 m), while in the highlands (approx. 1000 m), the temperature can reach 20 °C. Precipitation levels are high, close to or above 2500 mm [41].

Outside the protected areas of the study region, forest remnants are small and widely dispersed or consist of riparian forests that are gradually being reduced in size. Many forests in heavily populated areas have been destroyed. At the edges of rivers, there are gallery forests with medium sized trees and scrub/bushes. In areas of gently rolling hills, such as in LP, although the soil drainage is good, the quality of the soil is poor for agricultural production. In mountainous areas, such as in T, there are often very steep, more hilly terrains, and the agricultural capacity in small valleys is good due to good internal soil drainage [41].

### 2.2. Sand Fly Sampling

Sand flies were collected using Centers for Disease Control (CDC) type light traps (BioQuip, Rancho Dominguez, CA, USA). In each plot, we deployed a total of 12 traps in four 150 m long transects (Figure 1). In each transect, three traps were located every 50 m, and the distance between transects varied between 50 and 150 m. The specific location of each trap at LP is shown in Figure 1C. Trap locations for T are shown in Figure 1D. Sampling was done twice during the rainy season, in October 2013 and November 2014 at LP, and November 2013 and December 2014 at T, and twice during the dry season, February 2014 and March 2015 at LP and March 2014 and April 2015 at T. Each sampling session consisted of three consecutive overnight collections done at each plot. Traps were set between 6:00 pm and 6:00 am to coincide with peak sand fly activity [21,42,43] and hung from trees at a uniform height of 1.5 m. Sand flies collected by each trap were then placed in vials with 70% ethanol for conservation and posterior identification.

### 2.3. Sand Fly Species’ Identification

Sand flies were examined and separated under a Leica (Z30V model) dissection scope. Individual sand flies were then mounted on glass slides with Hoyer’s medium and examined after 24 h., allowing for clarification of internal and external morphological structures [19] for taxonomic identification. Phlebotomine sand flies were identified based on the morphology of male and female genitalia, head, cibarium, and wings using the taxonomic keys by Young and Duncan [44] and Galati [45]. We then classified sand flies according to their feeding habits as zoophilic or anthropophilic to separate species according to their medical importance [12].

### 2.4. Statistical Analysis

We estimated the total number of species for each plot, study location, and season using the Chao2 estimator [46] and then verified these estimates using species accumulation curves built by rarefaction [47], which are expected to flatten as most species in a given habitat are sampled. Following Chaves and Añez [18,19], we analyzed sand fly species’ co-occurrence patterns across the sampling plots using the C-score and the nestedness metric based on overlap and decreasing fills (NODF). Briefly, these indices indicate whether species segregate (or aggregate) when the C-score is above (or below) what is expected by random, and the NODF can tell if the aggregation is due to species’ nestedness, i.e., due to some plots having more species than others (using the NODF-Location index) and whether those patterns have some degree of species turnover (using the NODF-Species index). C-scores and NODFs were estimated using the program co-occurrence [31,32,48,49]. Inferences for the C-Score and NODFs indices were based on null model tests, where matrices were randomly generated assuming that species appeared with a constant probability, based on our observations, across sampling plots, but also assuming sand fly species sampling was equiprobable across the plots. Inferences for the C-score and NODF indices were based on 10,000 simulations, where *p*-values were based on the comparison of observed data and estimated indices against the distribution of values obtained from the randomly generated matrices [49].

We also compared species’ composition across plots and seasons using the Sorensen species’ similarity index, whose result was visualized through a hierarchical agglomerative cluster following the method described by Hoshi et al. [50]. We also tested if the differences between species, independent of season, were related to the geographic distance between the geographic center of the sampling locations at each plot using the multisite Sørensen, Simpson, and Nestedness indices [26,51]. These indices, respectively, allow testing for significant effects of geographic distance on dispersal, species’ turnover, and species decreasing nestedness as factors shaping differences in species’ composition [19]. We used a Pearson correlation test to estimate associations between species’ dissimilarity and geographic distance. To make statistical inferences, we did a 999 randomization Mantel test for the estimated Pearson correlation between each index and geographic distance, which is a powerful test with a minimum of five study points, i.e., plots in the context of our study [19]. For other details about the software employed for the analysis, please refer to Chaves and Añez [19].

## 3. Results

Between 2013 and 2015, 16,156 sand flies were collected in the communities of T (n = 6288, male/female: 2203/4085) and LP (n = 9868, male/female: 3858/6010). Taxonomic identification showed a total of 15 genera and 35 species of sand flies, with a total sampling effort of 710 trap-nights, as 10 trap-nights of sampling effort were lost at site T2 during the wet seasons of 2014 and 2015 by factors outside of our control (Table 1).

We identified a total of 34 species in T, and the most abundant species collected was *Ny. trapidoi* (37%), followed by *Ps. panamensis* (18%) and *Ty. triramula* (17%). The remaining species accounted for 28% of sand fly samples (Table 1). In LP, we identified 26 species, and the most abundant species collected were *Ty. triramula* (48%) and *Ps. panamensis* (35%), with the other species representing 17% of sand fly samples (Table 1).

During the wet season, in T, the most dominant species were *Ny. trapidoi* (46%) and *Ps. panamensis* (24%). In LP, the most abundant species were *Ps. panamensis* (71%) and *Ty. triramula* (16%). In the dry season, the most abundant species in T was *Ty. triramula* (51%), followed by *Lu. gomezi* (10%), *Pi. ovallesi* (8%), and *Ny. trapidoi* (8%). In LP, the most abundant species was *Ty. triramula* (79%), followed by *Pr. dysponeta* (8%) and *Lu. gomezi* (7%).

**Figure 2:**
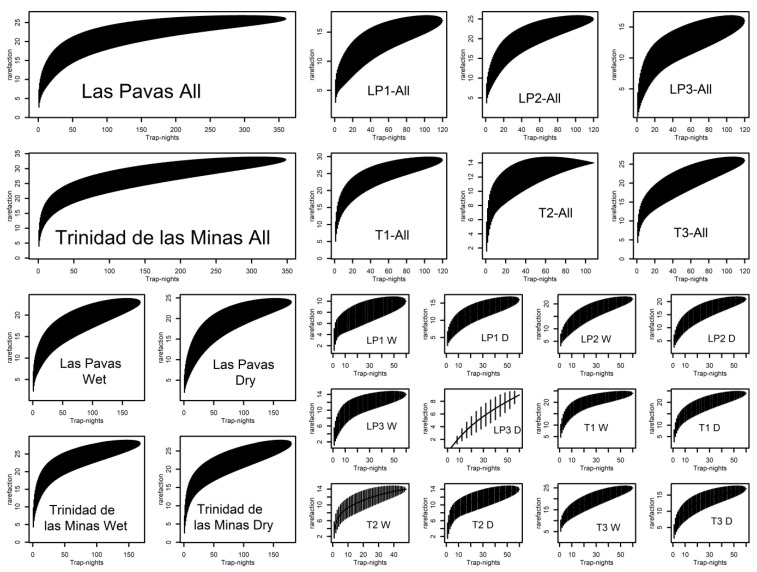
Species’ accumulation curves of the total number of sand fly species collected in the two study communities Las Pavas (LP) and Trinidad de las Minas (T) through the study period (All); and three plots from each community through the study period (LP1-peridomicile-All, LP2-partly wooded-All, LP3-wooded-All and T1-wooded-All, T2-peridomicile-All, T3-partly wooded-All); in LP and T during the Wet and Dry seasons; in each plot from each community during the wet (W) and dry (D) seasons.

In terms of species’ diversity, we observed the highest diversity index in T (Chao2 ± S.E. index: 36.58 ± 3.84), compared to LP (27.49 ± 2.28). The highest number of species in T were observed in the wooded (37.92 ± 10.09) and partly wooded plot (29.57 ± 3.82); the peridomicile plot showed less diversity (14.01 ± 0.01). In LP, the wooded and partly wooded plot showed a diversity index of 28.40 ± 17.00 and 26.59 ± 2.15, respectively, while the peridomicile plot presented a diversity of 19.64 ± 3.46. The species’ accumulation curves suggested that for LP and T, diversity was thoroughly sampled as the species’ accumulation curves for samples from all the study periods flattened (Figure 2), but not for some of the individual plots, specifically LP3 and T3, which did not flatten (Figure 2).

In relation to season, T showed different Chao 2 index values for dry and wet seasons (39.18 ± 13.08 and 31.11 ± 3.64, respectively). Diversity in LP, according to dry and wet season, was similar (26.49 ± 2.94 and 26.11 ± 3.64, respectively). The species’ accumulation curves suggested that for both LP and T, diversity was thoroughly sampled as the species’ accumulation curves for samples from each season flattened (Figure 2).

We compared the diversity by land use type according to season in the two communities. In T, there was an increase in diversity in the wooded plot from dry (29.90 ± 6.38) to wet season (41.70 ± 23.23). The partly wooded plot did not have sand fly diversity differences between dry (29.29 ± 16.86) and wet (29.82 ± 4.77) seasons. However, there was a reduction in sand fly richness at the peridomicile plot from dry (21.87 ± 11.47) to wet season (15.47 ± 2.25). In LP, the wooded plot did not show changes in diversity from dry (17.85 ± 10.01) to wet season (18.42 ± 7.07). In the partly wooded plot, we observed a small difference from dry (33.04 ± 12.94) to wet season (27.90 ± 6.38), and a drastic change from dry (33.70 ± 23.23) to wet season (14.42 ± 7.07) in the peridomicile plot. The species’ accumulation curves suggested that for LP and T diversity was thoroughly sampled only in LP1, LP2, T1, and T2 in both seasons, LP3 during the wet season, and that species sampling was incomplete in LP3 during the dry season and in T3 during both seasons (Figure 2).

The C-score analysis results are presented in Table 2. Based on C-score null models, we found that co-occurrence patterns of sand fly species in both study sites (LP and T) were significantly different between estimated and simulated C-score values (0.23 vs. 0.64, *p* < 0.05). We observed the same pattern for data collected during wet season (0.13 vs. 0.53, *p* < 0.05) and dry season (0.35 vs. 0.64, *p* < 0.05).

The results of the C-score analysis showed that in all cases, the estimated C-scores were smaller than the simulated values, indicating that sand fly species were aggregated. Since all three data sets showed aggregation patterns of sand fly species’ coexistent, we did a nestedness analysis (NODF) for each data set. The results for the NODF estimates are presented in Table 2.

Estimates of NODF-global (LP and T) were significantly higher than simulated (76.25 vs. 67.71, *p* > 0.05), indicating that sand fly communities were nested independently of the land use type (wooded, partly wooded, and peridomicile). Similarly, NODF-global analysis according to season (dry and wet) showed estimated NODF values higher than simulated (wet: 57.55 vs. 50.15, *p* < 0.05 and dry: 52.48 vs. 46.70, *p* < 0.05).

The NODF-locations, i.e., for land use plots (wooded, partly wooded, and peridomicile), were significantly larger than those expected by chance (92.58 vs. 71.18, *p* < 0.05), similarly when comparing the estimated vs. simulated according to season (wet: 88.89 vs. 69.47, *p* < 0.05 and dry: 86.38 vs. 66.57, *p* < 0.05).

**Figure 3:**
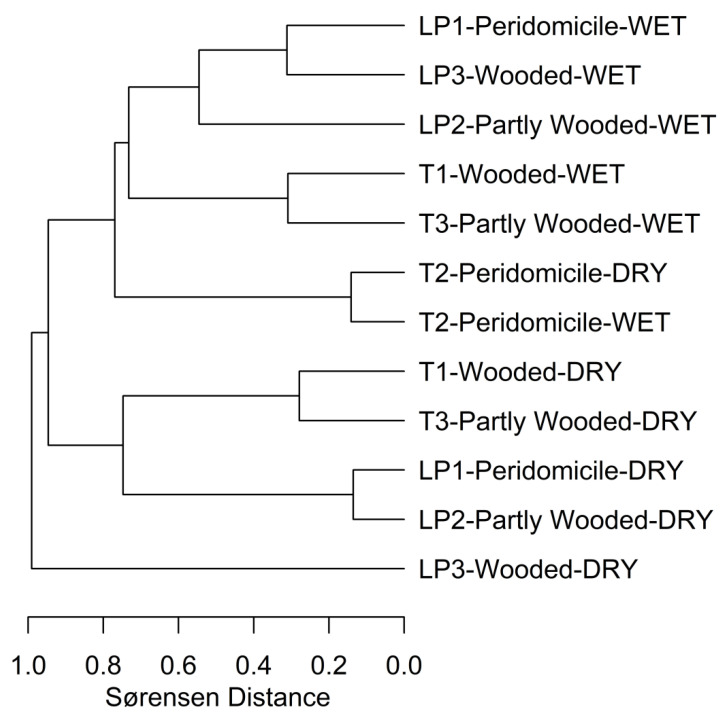
Cluster analysis of the Sørensen’s dissimilarity index for each plot and collection season. Labels stand for the following: Season collected: DRY (dry season); WET (wet season); Wooded; Partly Wooded, and Peridomicile plots are indicated in the labels, where LP (Las Pavas) and T (Trinidad de las Minas) codes correspond to the locations of Figure 1.

The NODF-species was significantly higher than expected by chance (75.84 vs. 67.62, *p* < 0.05), a result that supports a certain degree of species’ turnover. Similar results were observed comparing the wet and dry season (56.76 vs. 67.62, *p* < 0.05 and 51.62 vs. 46.20, *p* < 0.05). These results are illustrated by a Sørensen cluster analysis of dissimilarity when the results of the different plots of both communities are used (Figure 3). In this analysis, a wooded plot of LP (LP3) had the lowest sand fly species’ richness in the dry season, and the other plots exhibited a partial degree of species’ turnover.

We did not observe significant differences in sand fly species collected in relation to land use plots by analyzing data with a cluster analysis of Sørensen’s dissimilarity index (r multisite Sørensen: −0.1109, *p* = 0.56, r nestedness: −0.1154, *p* = 0.62).

## 4. Discussion

In this study, we characterized the diversity and abundance of sand flies in two rural areas (LP and T) of Western Panamá. We compared diversity by land use type (categorized in wooded, partly wooded, and peridomicile plots) and season (dry and wet season). In Latin America, it has been asserted that profound changes caused by the destruction of ecosystems by natural phenomena or human activity, such as deforestation, and changes in vegetation patterns have caused some sand fly species’ disappearance, while others have become more abundant or adapted to synanthropic environments, often modifying their behavior [52,53].

We observed high diversity of sand fly species in both rural areas, including zoophilic and anthropophilic species. Most sand fly species were captured in environments dominated by forest cover, which was expected because most of the sand fly species are associated with well-differentiated habitats [20,21,22,23,42,43,54,55]. Similar studies carried out in different areas of Manaus forests also found high sand fly species’ richness [56].

The 35 phlebotomine species identified included six confirmed *Leishmania* spp. vectors in Panamá, which include, *Ny. ylephiletor*, *Lu. gomezi*, *Lu. sanguinaria*, *Ps. thula*, *Ny. trapidoi*, and *Ps. panamensis* [17,57,58,59,60,61,62]. In both communities, the most abundant sand fly species were the anthropophilic *Ny. trapidoi* and *Ps. panamensis* and the zoophilic *Ty. triramula*. The presence of these vectors in different habitats and sampled areas shows the versatility and dispersal capacity of these species and their adaptation to highly human-disrupted habitats [63,64].

The anthropophilic species were abundant during the wet season in both communities (T and LP). However, we observed a switch since the zoophilic species *Ty. triramula* was the most abundant in both communities in the dry season. Phlebotomines are susceptible to habitat changes, yet this susceptibility is species specific and can have consequences for vector-parasite interactions [52,65]. For example, some studies have shown that vector and preferred or alternative hosts may vary in space and time, and for phlebotomine sand fly species vectors of leishmaniasis, it is well known that they generally bite various host species [14,44]. Thus, anthropophily may be represented as a complex nonlinear function of vectors and host abundance [66,67] instead of a simple dichotomic discrimination into “anthropophilic” or “zoophilic” species [18].

In general, sand fly diversity decreased due to the destruction of habitats, and several species were absent from the degraded area (peridomicile). Nevertheless, several species persisted in the disturbed habitats, albeit at lower abundances [52]. *Ps. panamensis* is one of the most successful species adapting to degraded habitats. *Ps. panamensis* and *Lu. gomezi,* found at both study sites, are anthropophilic sand flies considered to be major vectors across South America [52,68]. Additionally, it is worth noting that total females in the wet season represented 60% in LP and 68% in T.

Considering the analysis according to land use type, in T, there was a reduction in species’ richness (calculated by Chao2 index) from wooded, partly wooded, and peridomicile plots. In LP, wooded and partly wooded had similar results compared to peridomicile that showed lowers values in diversity. These striking regional differences in diversity could also reflect diversity differences associated with biogeographic barriers [69]. T, located in the Capira district (Figure 1), is an area markedly influenced by the Central Mountain Range, a mountainous formation that comes from Costa Rica and forms the backbone of the Panamanian relief. It constitutes a mountainous arch that extends from Costa Rica to Cerro Trinidad with an altitude of over 1000 m [41], which could serve as shelter for sand flies. On the contrary, LP, located in the Chorrera district (Figure 1), a lowland located on the west bank of the Panamá Canal, is an area currently consisting of patches of forest remnants located mainly in riparian areas [34].

Patterns of coexistence of species at the local scale could change with landscape transformation. These changes could also impact the dynamics of *Leishmania* spp. transmission, especially if the changes are coupled with changes in the composition of reservoir and incidental hosts [70]. In addition, the change in land use can modify the composition of the vector community. Sand fly species with greater ecological fitting, that is, species with the ability to use new or transformed habitats [71], can dominate communities that used to be very diverse before land use change occurred. It is expected that undisturbed environments, such as forests, when compared with transformed landscapes, such as farmland or peridomiciles, have more structured communities with regard to their choice or use of resting sites, which are of great importance for risk of leishmaniasis transmission, given their limited dispersal [54,72]. Furthermore, it is possible that regional land use could exacerbate seasonal differences in microclimate impacting sand fly populations [73,74]. This might explain why there was no seasonal difference in species’ richness in LP, but, in contrast, species’ richness increased in T, an area with larger forest patches than LP.

We can also affirm that our analysis is based on a set of high-quality data, with a systematic and standardized sampling effort [75]. The patterns of sand fly species’ accumulation, irrespective of sampling plots, suggest that sites housing the largest number of species could have more diverse microhabitats and possibly host communities that support a greater diversity of sand flies [18] than what we measured.

Although the study communities LP and T are relatively close to one another, they differ in the patterns of relative abundance of sand fly vectors of American cutaneous leishmaniasis (ACL) [76].

The partial replacement of species detected by the NODF analysis is related to rare species that were only found in most of the species-rich sites, specifically *Pa. abonnenci*, *Pa. aclydifera*, *Vi. caprina*, *Lu. cruciata*, *Pa. dasymera*, *Ev. dubitans*, *Lu. longipalpis*, *Pi. odax* were only collected in T, and only two species, *Ps. geniculatus* and *Pa. punctigeniculata,* were collected in LP but not in T. Interestingly, six species of medical importance were common in all sampling sites: *Ny. trapidoi*, *Ps. panamensis*, *Ny. ylephiletor*, *Lu. gomezi*, *Lu. sanguinaria*, and *Ps. thula.*

Two species not known to have medical importance in Panamá, but reported to be *Leishmania* vectors in Belize, Colombia, Ecuador, Guatemala, Mexico, and Venezuela, were *Bi. olmeca bicolor* [77,78] and *Pi. ovallesi*. In Panamá, *Pi. ovallesi* is considered a ‘potential’ vector because it was found infected with unidentified flagellates [12] and was common at most sampling sites, a pattern also observed in Venezuela [79].

We did not observe significant differences in sand fly species collected in relation to land use plots distance after analyzing data with cluster analysis of Sørensen’s dissimilarity index. Thus, any potential impact that dispersal limitation could have on species’ turnover was not observed in our results [26]; since there were no differences in species of sand fly fauna collected in the different plots of both communities, there were no patterns influenced by the distance between sampling locations.

## 5. Conclusions

Habitat degradation negatively affected species’ richness in sand fly communities at our study sites. Seasonal differences are particularly important determinants of sand fly relative abundance, species’ richness, and species’ composition. However, medically important species were able to exploit, indeed have higher abundance, in modified environments, and might contribute to *Leishmania* spp. endemicity. Going forward, it is important to understand the possible association between species’ composition, nestedness, hybridization capacity [80], and the phylogenetic relationships of sand fly species [81], paying special attention to the fact that *Leishmania* spp. vector species often tend to be more widespread than species without medical importance.

## Figures and Tables

**Table 1:** Abundance and diversity of sand flies collected from 2013 to 2015 in Las Pavas (LP) and Trinidad de las Minas (T), Western Panamá.

Location	LP			T		
Species	F	M	Total	F	M	Total
*Brumptomyia hamata* (Fairchild and Hertig, 1947)	0	1	1	0	2	2
*Bichromomyia olmeca bicolor* (Fairchild and Theodor, 1971)	19	6	25	126	63	189
*Dampfomyia vesicifera* (Fairchild and Hertig, 1947)	4	0	4	25	2	27
*Dampfomyia vespertilionis* (Fairchild and Hertig, 1947)	7	2	9	8	9	17
*Evandromyia dubitans* (Sherlock, 1962)	0	0	0	1	0	1
*Evandromyia saulensis* (Floch and Abonnenc, 1944)	4	0	4	3	0	3
*Lutzomyia longipalpis* (Lutz and Neiva, 1912) **	0	0	0	2	0	2
*Lutzomyia cruciata* (Coquillett, 1907)	0	0	0	1	0	1
*Lutzomyia gomezi* (Nitzulescu, 1931) *	369	213	582	134	88	222
*Lutzomyia sanguinaria* (Fairchild and Hertig, 1957) *	3	1	4	10	2	12
*Lutzomyia sp* (França, 1924)	1	10	11	22	5	27
*Micropygomyia micropyga* (Mangabeira,1942)	3	13	16	0	5	5
*Micropygomyia trinidadensis* (Newstead, 1922) **	1	2	3	14	33	47
*Nyssomyia trapidoi* (Fairchild and Hertig, 1952) *	114	38	152	1684	594	2278
*Nyssomyia ylephiletor* (Fairchild and Hertig, 1952) *	7	9	16	260	208	468
*Pintomyia odax* (Fairchild and Hertig, 1961)	0	0	0	1	0	1
*Pintomyia serrana* (Damasceno and Arouck, 1949)	3	0	3	34	17	51
*Pintomyia ovallesi* (Ortiz, 1952) **	15	7	22	215	59	274
*Pressatia camposi* (Rodríguez, 1950)	97	38	135	39	45	84
*Pressatia dysponeta* (Fairchild and Hertig, 1952)	293	195	488	80	45	125
*Psathyromyia abonnenci* (Floch and Chassignet, 1947)	0	0	0	1	0	1
*Psathyromyia dasymera* (Fairchild and Hertig, 1961)	0	0	0	2	0	2
*Psathyromyia punctigeniculata* (Floch and Abonnenc, 1944)	2	0	2	0	0	0
*Psathyromyia runoides* (Faichild and Hertig, 1953)	0	2	2	4	1	5
*Psathyromyia aclydifera* (Fairchild and Hertig, 1952)	0	0	0	18	8	26
*Psathyromyia carpenteri* (Fairchild and Hertig, 1953)	12	12	24	10	5	15
*Psathyromyia shannoni* (Dyar, 1929) **	6	1	7	2	4	6
*Psychodopygus geniculatus* (Mangabeira,1941)	1	0	1	0	0	0
*Psychodopygus panamensis* (Shannon, 1926) *	2104	1340	3444	653	459	1112
*Psychodopygus thula* (Young, 1979) *	61	107	168	113	90	203
*Sciopemyia sordellii* (Shannon and Del Ponte, 1927)	10	5	15	8	3	11
*Trichopygomyia triramula* (Fairchild and Hertig, 1952)	2874	1855	4729	611	452	1063
*Viannamyia caprina* (Osorno-Mesa, Morales and Osorno, 1972)	0	0	0	2	0	2
*Viannamyia furcata* (Mangabeira, 1941)	0	0	0	1	4	5
*Warileya rotundipennis* (Fairchild and Hertig, 1951)	0	1	1	1	0	1
**Total**	6010	3858	**9868**	4085	2203	**6288**

F is for females and M for males. * Confirmed vector in Panamá. ** Confirmed vector elsewhere in Latin America.

**Table 2:** C-score and nestedness overlap and decreasing fills (NODF) for sand fly species sampled at different land use plots and season in the Las Pavas and Trinidad de las Minas communities.

Metric	Sampling	Estimated	Mean Simulation	95% CI
C-score	C-score both	0.23 *	0.64	(0.54, 0.71)
	C-score wet	0.13 *	0.53	(0.44, 0.59)
	C-score dry	0.35 *	0.64	(0.54, 0.71)
				
NODF-Global	NODF-both	76.25 *	67.71	(65.85, 69.98)
	NODF-wet	57.55 *	50.15	(48.59, 52.17)
	NODF-dry	52.48 *	46.70	(45.02, 48.83)
				
NODF-Locations	NODF-both	92.58 *	71.18	(49.51, 83.30)
	NODF-wet	88.89 *	69.47	(48.19, 82.21)
	NODF-dry	86.38 *	66.57	(46.48, 78.25)
				
NODF-Species	NODF-both	75.84 *	67.62	(65.94, 69.75)
	NODF-wet	56.76 *	49.67	(48.29, 51.48)
	NODF-dry	51.62 *	46.20	(44.57, 48.18)

* Statistically significant (*p* < 0.05).

## Data Availability

Data are available upon reasonable request.
